# Ground beetle (Coleoptera, Carabidae) assemblages inhabiting Scots pine stands of Puszcza Piska Forest: six-year responses to a tornado impact

**DOI:** 10.3897/zookeys.100.1360

**Published:** 2011-05-20

**Authors:** Jarosław Skłodowski, Paulina Garbalińska

**Affiliations:** Department of Forest Protection and Ecology, Warsaw University of Life Sciences, Nowoursynowska 159, 02-776 Warszawa, Poland

**Keywords:** carabid beetle, disturbance, assemblage structure, Mean Individual Biomass, Sum of Progressive Characteristics, forest fauna

## Abstract

Ground beetle assemblages were studied during 2003-08 in the Pisz Forest by comparing stands disturbed by a tornado to undisturbed control stands. The following exploratory questions were put forward. (1) How do the carabid assemblages change during six years following the tornado impact? (2) Does the carabid assemblage recovery begin during the six first post-tornado years? To assess the state of carabid assemblages we used two indices: the MIB (Mean Individual Biomass) and the SPC (Sum of Progressive Characteristics). Carabid assemblages in the disturbed and in the control stands, as expressed by these two indices, were compared using the length of a regression distance (sample distance in a MIB:SPC coordinate system). A cluster analysis revealed that the assemblages of the disturbed and the control stands were different. The tornado-impacted stands produced lower carabid catch rates, but species richness was significantly higher there than in the control stands. They hosted lower proportions of individuals of European species, of large zoophages, and of forest and brachypterous species, than the control stands. The observed reduction in SPC and MIB, and an increase in the regression distances may indicate that the carabid assemblages had not started to recover from the tornado-caused disturbance. Carabid assemblages apparently responded to the tornado in two steps. Firstly, the first three years were characterized by moderate decreases of index values. Secondly, from the fourth to the sixth year after the tornado, many observed changes became magnified. We did not observe clear signals of the recovery of forest carabid assemblages during the six follow-up years.

## Introduction

Natural disturbances of ecosystems are often short term but characterized by high amplitude. Disturbances are influential for the dynamics of both structure and functioning of ecosystems, and are their integral part. They vary from large scale (e.g., wildfire or hurricane) to local ones that may concern only single or small groups of trees (Pickett and White 1985; Faliński 1986; Pontailler et al. 1997; Bengtsson et al. 2000; Szwagrzyk 2000; Chapin et al. 2002; Wolf et al. 2004).

Following a disturbance, such as a tornado impact, forest stands (here, stand is a patch with a cluster of dominant trees of the same origin, e.g., initiated by a clear-cutting event, and that is surrounded by patches of other types of habitat or trees of different origin) consist of a mosaic of patches impacted to varying degrees, from completely destroyed trees to remnants of untouched forest. The heterogeneity and diversity of microhabitats rapidly increase. In these stands, patches of barren soil, directly exposed to the drying effect of the sun and wind, may be abundant. Under such conditions, the structure and composition of forest-floor vegetation are obviously altered: the soil becomes drier, and the ground surface may be covered by fallen trees and other organic material subject to slow decomposition (Ulanova 2000; Bouget and Duelli 2004; Bouget 2005a, 2005b; Skłodowski 2007a). Such dramatic changes in the environmental conditions in turn alter invertebrate assemblages, including carabid beetles (Bouget 2005c). However, the speed and duration of these alterations are poorly understood.

In the short term, a tornado-caused disturbance changes the functioning and structure of the forest ecosystem. The lack of canopy shelter triggers a regression process (here, a decrease in the number of species associated with closed forests and a simultaneous increase of open-area species), but the emergence of tree saplings starts at the onset of the regeneration succession. The regeneration of a disturbed ecosystem may be faster due to the mosaic-type spatial pattern. For example, under mosaic-like spatial conditions, the emergence and subsequent growth of Scots pine (*Pinus sylvestris*) saplings takes place through the seed pool accumulated in the soil before the disturbance and also through the seed fall from the survived trees. Similarly, stands not impacted by the disturbance may act as sources of organisms recolonizing the disturbed stands.

Carabid beetles have been extensively studied in various, disturbed forest ecosystems (e.g., Ings and Hartley 1999; Bouget 2005c; for a review, see Niemelä et al. 2007). Most studies on carabids in disturbed stands usually cover only the first 2-3 years after the disturbance and – most likely because of this short-term nature – have not revealed information on how quickly carabid assemblages might recover after the disturbance (Bouget 2005c). Follow-up studies lasting more than a couple of years would identify the onset of carabid assemblage recovery.

A unique opportunity to follow disturbance-initiated changes in forest-carabid assemblages over a long time period appeared in July 2002, when a tornado destroyed 33,000 hectares of Scots pine forest in north-eastern Poland. The majority of the damaged stands were soon cleared and planted with pine saplings. However, an untouched area of about 445 ha (“Szast Protective Forest”) was left unmanaged. A research project on the tornado impact started in 2003 (Skłodowski and Zdzioch 2005; Skłodowski 2007b, 2007c; Skłodowski and Garbalińska 2007a, 2007b) and continues to date. In the present paper, the following exploratory questions were put forward, using data on the first six post-tornado years: (1) How do carabid assemblages change during the first six years after the tornado-caused disturbance? In particular, we predicted that (a) large forest species with low dispersal power would be less abundant in the disturbed stands, compared to undisturbed control stands, and that (b) non-forest species with good dispersal potential would show the opposite. (2) Does the carabid assemblage recovery begin during the first six years after the disturbance? Earlier Niemelä et al. (1993) showed that carabid assemblages had partially recovered from clear-cut harvesting after a 25-30 years time span in Canada.

## Study area and methods

The present study was done in Scots pine forests growing on podzol soils in the Pisz Forest District area, in post-tornado (disturbed stands; Szast Protective Forest) and in intact stands (control; Maskulińskie Forest District). The two study areas were located 20 km apart in order to exclude any influence of tornados in the control stands. The two study areas both hosted three replicate stands (study plots) for each of the following five age classes: 20-40 years old (class I), 40-50 (class II), 50-60 (class III), 60-80 (class IV) and >80 years old (class V). We thus had a total of 15 stands in the disturbed area (‘disturbed stands’) and 15 in the control area (‘control stands’), making up altogether 30 stands (plots).

Five pitfall traps (0.5 l glass jar with a plastic funnel, 12 cm in diameter, containing 100 ml 70% ethylene glycol, and covered by a 20 cm x 20 cm roof a few cm above soil surface to protect the samples against rain and litter) were arranged 15 m apart along a transect in each study stand. The traps were continuously operating between early May and the end of October, and serviced every six weeks. For each carabid individual caught, body length was measured with a microscope and with accuracy of 0.5 mm (from the top of mandibles to the tip of elytra) in order to calculate their biomass following Szujecki et al. (1983).

All specimens captured were identified to species level. The collected data were pooled separately for each stand. A standardized catch rate (individuals/day*trap) was calculated to account for occasional trap losses. The following measures were subjected to analysis: standardized catch rates for each species and individuals, species richness, and the proportion of individuals belonging to various functional groups. For the latter purpose, species were grouped according to geographic distribution (inhabiting European or Palearctic and Holartic regions), habitat association (forest and non forest species), food preference (large zoophages, small zoophages and hemizoophages) and wing morphology (brachypterous species and macropterous species). Species richness was standardized to the lowest number of specimens in the samples using rarefaction (Krebs 1999).

Moreover, two indices were calculated for the total catch: Mean Individual Biomass (MIB; e.g., Szyszko 1990, 1997) and the Sum of Progressive Characteristics (SPC; Skłodowski 1995, 1997, 2009). Both indices are positively correlated with stand age: for MIB, see (Szyszko (1983, 1997) and (Skłodowski (1995, 2002, 2006a, b), and for SPC, see (Skłodowski (1997, 2006a, b, 2009). The latter is calculated as follows:

SPC = 74.9 + 102 * LOG (stand age) (1)

The two values in the formula (1) are model coefficients: 74.9 for the intercept and 102 for the regression slope. SPC is the sum of proportions of species associated with successional “old-growth” stands over all sampled stands. These species are, in particular, large, zoophagous, European forest carabids with autumn development (Skłodowski 1995, 1997, 2002, 2006a, 2009). In assessing the state of carabid assemblages these indices are complementary because they have different characteristics (dynamics) with the age of the forest: MIB increases more rapidly in stands older than 20 years, while SPC increases rapidly somewhat later, 40 years after the initiation of secondary succession.

The MIB and SPC indices can be presented in an X-Y coordinate system to produce an SPC/MIB model that efficiently summarizes the successional development status of species assemblages (Skłodowski 1997, 2009). Carabid assemblages inhabiting old-growth stands scatter in the upper right-hand corner of such a graph, while assemblages of clear cut areas (recent, severe disturbance) tend to be located down and to the left. In this coordinate system, the distance between the old-growth stands and newly-planted forest cultures reflects the so-called “regression distance” that may be calculated using the cosine rule (Skłodowski 1995, 1997, 2009). As a regression of carabid assemblages we assume transformation of functional structure of assemblages characterized by high level of ecological successive development (ex. inhabiting old growth stands) into assemblages characterized by low level of successive development (ex. inhabiting clear cut areas or young coppices). This means an increase of species number due to colonization of open habitat species and a decline of forest species. Also the participation of individuals belonging to big zoophageous species decreases while the participation of small hemizoophageous, non forest species with high dispersal powers, increases in the assemblages. The angle at which a vector connecting extreme points is directed reflects the trend of the change, being either regressive or regenerating (Skłodowski 1995, 1997, 2009). By calculating the square root of the product MIB×SPC, one obtains an average value for MIB:SPC coordinate pairs, thus producing a value that reflects the developmental phase of carabid assemblages, which can be tested statistically. The difference between the roots of the product MIBxSPC in assemblages of the disturbed and control stands gives a regression distance between the two stand types. In other words, the regression distance shows a difference between two phases of succession that can be expressed as a distance between two compared assemblages plotted on a graph.

Prior to the statistical analyses, data were tested for the normality of distributions and variance homogeneity using Shapiro-Wilks and Levene’s statistics (Statistica; StatSoft Inc. 2008). For data with equal variances and normal distribution, ANOVA with repeated measures was applied to assess the similarity between disturbed and control stands for most comparisons. However, Mann-Whitney U test was used to examine the proportion of individuals of forest species due to a lack of data normality.

The dependence of carabid recovery in time since the tornado was tested using ANOVA with repeated measures (if normality and variance homogeneity assumptions were fulfilled) or Mann-Whitney U test (Statistica; StatSoft Inc. 2008). We verified the impact of the tornado on the proportion of individuals of European species, on MIB and SPC, as well as on the regression distances. We thus compared two stand types: disturbed versus control, both with nested plots of five age classes (I–V), each class being replicated three times for both stand types. The six years (2003-2008) defined the repeated measures. Subsequent pair-wise comparisons for significant differences were done using the least significant difference (LSD) *post hoc* test. Mann-Whitney U test was used to verify the impact of the tornado on the total catch rate, number of species, proportion of individuals of forest and eurytopic species, large and small zoophages, hemizoophages, and brachypterous and macropterous species. We examined compositional similarities among stands of different age and habitat type (disturbed vs control) using Ward cluster analysis based on Euclidean distances (Statistica; StatSoft Inc. 2008).

## Results

During the first six years following the tornado impact, the disturbed stands produced a total of 18,022 individuals belonging to 82 species, while the control stands produced 19,550 individuals representing 53 species. Altogether these made up 37,572 specimens representing 90 species. The catch rate over all years was 0.18 individuals/trap*day in the disturbed and 0.26 individuals/trap*day in the control stands (Z = 5.29; p < 0.001). This difference was statistically significant for the years 2003-2006. During 2007-2008 the catch rate of carabids in the disturbed stands was marginally higher than in the control (Table 1).

**Table 1.** Mann-Whitney U test for the total carabid catch rate (number of individuals/trap*day), rarefaction standardized species richness, and the proportion of individuals of different ecological, trophic and dispersal groups of carabids. Disturbed (D) and control (C) stands were compared during 2003-2008; test statistics (U), statistical significance (asterisks), and mean and SD values for D and C are shown. Significance levels: *** – p < 0.001; ** – p < 0.01; * – p < 0.05; n.s. – p > 0.05.

	*2003*	*2004*	*2005*	*2006*	*2007*	*2008*
Catch rate						
U	1.929 *	4.646 ***	3.380 ***	3.733***	n.s.	n.s.
D	0.11±0.06	0.06±0.02	0.14±0.07	0.15±0.06	0.36±0.23	0.28±0.12
C	0.20±0.10	0.25±0.06	0.24±0.09	0.29±0.09	0.34±0.14	0.22±0.06
Species number						
U	4.314 *	3.380 ***	n.s.	3.443 ***	4.646 ***	3.899 ***
D	9.05±1.79	6.93±1.56	8.65±1.66	11.79±2.45	16.43±3.89	13.92±3.86
C	6.31±0.87	8.81±1.15	8.72±1.15	8.61±0.78	8.51±0.92	9.01±1.32
Forest species						
U	4.148***	3.795***	4.500***	4.646***	4.646***	4.646***
D	87.7±11.11	87.0±11.10	86.7±8.4	65.8±17.11	57.9±15.31	46,3±15.43
C	98.6±2.08	98.7±1.61	97.8±1.56	98.5±0.91	99,2±1.12	96,5±4.15
Eurytopic species						
U	2.966**	2.903**	4.653***	4.646***	4.646***	4.646***
D	7.1±8.17	5.9±5.33	8.2±5.95	23.4±15.78	34.5±13.23	47.7±15.94
C	1.2±1.55	1.1±1.58	0.5±0.71	0.5±0.64	0.6±0.87	2.5±3.95
Large zoophages						
U	1.494*	n.s.	n.s.	2.737***	2.115**	4.272***
D	60.3±17.11	63.4±16.49	72.7±12.4	43.0±21.21	45.7±17.67	33.2±11.46
C	71.8±10.89	67.2±8.88	66.2±11.74	63.0±10.33	59.8±13.51	63.7±12.84
Small zoophages						
U	n.s.	n.s.	3.526***	n.s.	3.650***	2.862**
D	31.3±14.58	26.6±12.55	17.4±7.18	30.2±11.88	19.6±10.49	21.4±11.11
C	28.1±11.04	32.7±8.82	33.6±11.76	36.6±10.19	39.6±13.27	34.3±11.75
Hemizoophages						
U	4.604***	3.899***	4.521***	4.646***	4.646***	4.646***
D	8.5±10.08	10.0±9.18	9.8±7.88	26.8±16.34	34.6±16.17	45.4±16.50
C	0.1±0.31	0.1±0.19	0.3±0.41	0.4±0.71	0.6±1.21	2.0±3.64
Brachypterous species						
U	n.s.	n.s.	n.s.	3.816***	4.134***	4.646***
D	72.5±15.78	65.3±17.07	78.4±11.02	58.4±16.75	54.3±15.71	42.1±12.72
C	75.2±9.52	68.9±8.56	80.9±7.38	83.2±5.00	85.6±5.18	79.6±7.62
Macropterous species						
U	n.s.	n.s.	n.s.	3.028**	3.892***	4.438***
D	21.5±13.53	31.1±16.37	20.3±11.25	33.2±17.20	37.5±16.00	50.0±15.88
C	23.5±8.60	29.8±8.78	18.6±7.43	16.3±5.00	14.0±5.06	19.1±7.13

The mean number of carabid species was greater in the disturbed than in control stands throughout the study: 11.13 *vs.* 8.33 (Z = 4.60, p < 0.001). During 2004-2005, the number of species was similar in the disturbed and control stands (Table 1) but during 2006-2008), the number of species was higher in the former, the difference being most pronounced in 2007. These observations reflect the ongoing process of species turnover in the disturbed stands: forest species decline and non-forest species and/or species absent from control stands increase, such as *Amara aenea*, *Amara commmunis*, *Amara equestris*, *Harpalus flavescens*, *Harpalus solitaris* and *Microlestes minutulus*.

**Figure 1.**
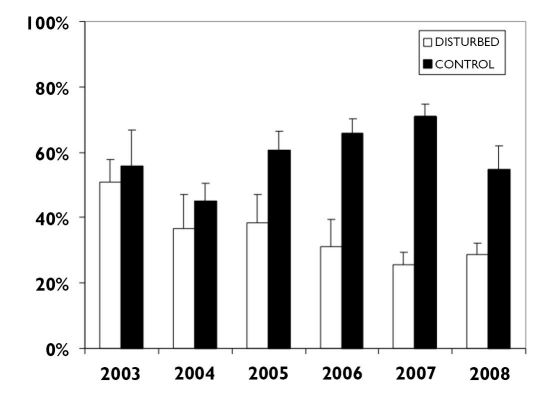
The proportion of individuals of European carabid species living in tornado-impacted and in control stands during 2003-2008.

During the six study years, the proportion of individuals of species with European distribution gradually decreased in the disturbed stands; as a consequence, the shares of Palaearctic and Holarctic species increased there (Fig. 1). Considering the entire six-years period, the average share of species with European distribution was 35.2% in the disturbed and 58.8% in the control stands (LSD test, p < 0.001). The difference in the proportion of individuals of European species between disturbed and control stands increased from 5.0% (55.8% vs. 50.8%) in 2003 to 45.3% (70.9% vs. 25.6%) in 2007 (Fig. 1).

The dominance structure of carabids changed from 2003 to 2008. In the disturbed stands in 2003, the Palaearctic *Carabus arvensis* (28.2%) was nearly equally abundant as the European *Carabus violaceus* (24.8%). However, in 2007, the Palaearctic *Carabus arvensis* and *Amara lunicollis* dominated, altogether making up 62.1% of the total catch in the disturbed stands, and 15.1% in the control stands.

During the six years of study, the proportion of individuals of forest carabids was on average 26.3% lower in disturbed than in control stands (71.9% *vs.* 98.2; Z = 10.74; p < 0.001). Until 2005, individuals of forest species were 87.7%-86.7% in the disturbed stands and 97.8%-98.7% in the control stands. Since 2006, the proportion of individuals of forest carabids in the disturbed stands decreased, reaching an all-time low of 46.3% in 2008. In the control stands for the same period, the proportion consistently remained around 96.5 % (Table 1). These differences can be seen at the species level too. In the disturbed stands, the dominant species during 2003-2008 was the forest dweller *Carabus arvensis* (up to 43.9 ± 15.1 % of the total catch in the disturbed and 26.1 ± 11.50 in the control stands). Simultaneously, the proportion of many other forest species gradually decreased: *Carabus hortensis*, *Carabus violaceus* and *Pterostichus oblongopunctatus* (Appendix A).

In the disturbed stands, forest species decreased and eurytopic species increased during the six study years. Eurytopic carabid individuals made up on average 21.1% of the catch in the disturbed and 1.1 % in the control stands (Z = 10.08; p < 0.001). During the first three years following the tornado, the proportion of individuals of eurytopic species in the disturbed stands varied between 5.9% and 8.2%; however, since the fourth year (2006), the proportion increased to, on average, 47.7% in 2008 (Table 1). The increase of eurytopic carabid individuals in successive years was significant (from 2005 to 2008; Z = 2.16 to 3.23, p = 0.028 to 0.001). Eurytopic species that dominated in the disturbed stands included *Amara lunicollis* (6.6%-36.7%), *Calathus erratus* (2.8%-4.8%) and *Harpalus rufipalpis* (3.3%-5.0%; Appendix B). Open-area species could also be found in the disturbed stands at a rather constant proportion of 5.1%-10.8%.

Considering the entire period of study, the proportion of individuals belonging to large zoophages in the disturbed stands was, on average, 12.2% lower than that in control stands (53.1% *vs.* 65.3%; Z = 3.93; p < 0.001). However, in 2004 and 2005 (second and third year after the tornado), the difference was not significant (Table 1). In the control stands in 2003-2005, large species dominated: *Carabus arvensis* (20.0%-24.9%), *Pterostichus niger* (20.3%-24.7%), *Carabus violaceus* (10.6%-16.8%) and *Carabus coriaceus* (5.4%-8.3%). In the disturbed stands, on the other hand, *Carabus arvensis* was the most numerous species (24.4-43.9% of the total catch), *Carabus violaceus* contributed 19.6%-25.4%, and *Pterostichus niger* made up 6.4%-7.7% there. During 2006-2008, the proportion of large zoophages was 14.0%-30.4% lower in disturbed than in control stands. During the six years of study, also the proportion of individuals of small zoophages decreased in the disturbed stands from 31.3% to 21.4% but increased in the control stands from 28.1% to 34.3% (Table 1).

The decreasing proportion of individuals of either large or small zoophages in the disturbed stands was accompanied by an increase of the hemizoophages; their proportion was on average higher in disturbed than in control stands (22.5% *vs.* 0.6%; Z = 10.84, p < 0.001). The disturbed stands had apparently been subject to colonization by hemizoophages since the first year after the tornado (2003), but the proportion of their individuals started to significantly increase in the fourth year after the tornado (2006). In the year 2008, individuals of hemizoophages contributed > 45% of all individuals in the disturbed stands (Table 1). The most frequent hemizoophage caught in disturbed stands was *Amara lunicollis* that dominated the catch in 2008 (36.7%), replacing *Carabus arvensis* (17.2%) as the most abundant species there (Appendix B).

During 2003-2008, the average proportion of individuals of the brachypterous species in the control stands was 78.9%, while in the disturbed stands it was 61.8% (Z = 6.14, p < 0.001). During the first three years of study (2003-2005), the difference in proportion of individuals of wingless carabids between disturbed and control stands was non-significant (Table 1). Since 2006, the proportion of individuals of brachypterous species decreased in the disturbed stands from 58.5% to 42.1%, and the difference between disturbed and control stands was 37.5% in 2008 (42.1% *vs.* 79.6%; Table 1). Wingless species decreased and macropterous species increases in the disturbed stands. Over the whole study period, the individuals of macropterous species made up 32.3% in the disturbed and 20.2% in the control stands (Z = 4.56, p < 0.001). The increase in the proportion of macropterous species in the disturbed stands apparently started in 2006, when the macropterous *Amara lunicollis* started to become abundant (Appendix B). Individuals of wing-dimorphic species became slightly more abundant in the disturbed stands, but never reached 10% during the study years (averages 5.9% for disturbed and0.9% for control stands; Z = 7.10, p < 0.001).

**Figure 2.**
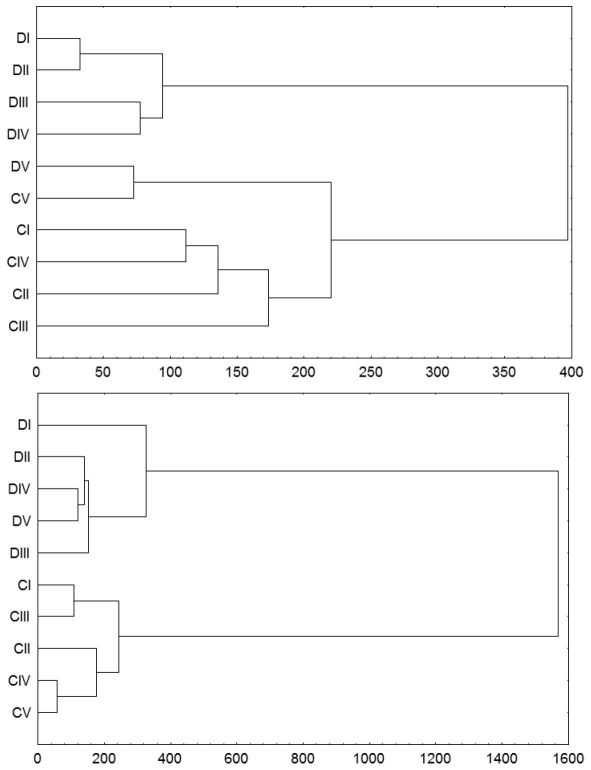
Dendrograms of species similarity of carabid beetle assemblages inhabiting tornado-impacted (D) and control stands (C) in age classes I–V (see text) in the first (2003) and last (2008) years of observation. The analysis was performed with the Ward method and Euclidean distance as the measure of similarity.

A cluster analysis (Fig. 2) for the carabid data similarity indicated faunal differences between disturbed and control stands, indicated by two stand clusters already in the first year after the tornado (2003). The analysis also lumped the oldest disturbed stand into the cluster of control stands. In the subsequent years, the carabid assemblages of the disturbed and control stands were distinctive, with decreasing similarity. Fig. 2 shows similarity diagrams for 2003 and 2008. These observations were somewhat supported by the indices: the Renkonen index for the similarity between disturbed and control stands was 64.4% in 2003 and 45.8% in 2008 (Table 3).

The response of carabid assemblages to the tornado disturbance could also be demonstrated using MIB and SPC indices and the regression distances retrieved from a SPC/MIB model (see Material and methods). The mean MIB during the six years of study was 214.3 mg for the disturbed and 303.0 mg for the control stands (LSD test; p < 0.001; Table 2).

**Table 2.** Repeated-measures ANOVA for the proportion of individuals of European species, for MIB and SPC indices, and for the regression distances describing the carabid assemblage of stands disturbed by the 2002 tornado, and in intact control stands. Factors: Stand (disturbed vs control stands), Age (stand age class I-V), and Time (2003–2008). LSD post hoc test explanation: 1-3 (2003-2004), 4-6 (2005-2006), D (disturbed stands), C (control stands), I-V (age class). Significance levels: *** – p < 0.001; ** – p < 0.01; * – p < 0.05; n.s. – p > 0.05).

*Effect*	*SS*	*df*	*MS*	*F*	*P*	*Post-hoc test*
*European sp.*						
Time	3422.2	5	684.4	7.82	<0.001	1-3 > 4-6**
Time*Stand	8879.0	5	1775.8	20.28	<0.001	D < C***
Time*Age	1576.4	20	78.8	0.90	n.s.	
Time*Stand*Age	1634.2	20	81.7	0.93	n.s.	
Error	8754.9	100	87.5			
*MIB*						
Time	694244.8	5	138849.0	48.03	<0.001	1-3 > 4-6***
Time*Stand	65289.1	5	13057.8	4.52	<0.001	D < C***
Time*Age	105863.3	20	5293.2	1.83	0.0269	I-III < IV-V**
Time*Stand*Age	56179.9	20	2809.0	0.97	n.s.	
Error	289092.1	100	2890.9			
*SPC*						
Time	69415.4	2	13883.1	20.60	<0.001	1-3 > 4-6***
Time*Stand	92119.1	2	18423.8	27.33	<0.001	D < C***
Time*Age	10586.2	20	529.3	0.79	n.s.	
Time*Stand*Age	11353.2	20	567.7	0.84	n.s.	
Error	67402.1	100	674.0	0.84		
*Regressive distance*						
Time	200832	5	40166	14.24	<0.001	1-3 < 4-6***
Time*Age	45559	20	2278	0.81	n.s.	
Error	141080	50	2822			

**Table 3.** Sørensen index for compositional similarity and Renkonen index for dominance structure for carabid assemblages inhabiting tornado-disturbed and intact control stands during 2003–2008 (% units).

*Index*	*2003*	*2004*	*2005*	*2006*	*2007*	*2008*
Sørensen	58.46	55.17	68.97	67.44	65.91	65.17
Renkonen	64.43	68.00	55.37	45.78	36.19	45.80

**Figure 3.**
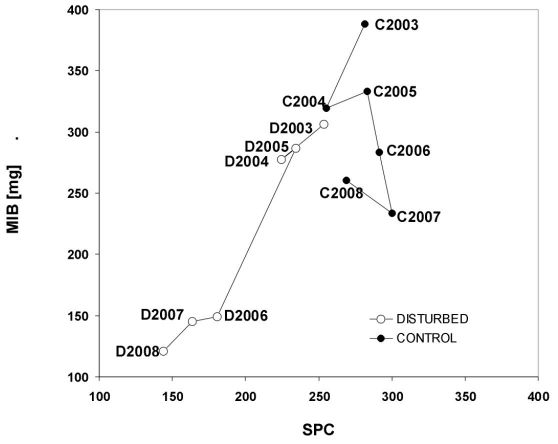
The MIB/SPC model of carabid assemblages living in tornado-impacted (D) and control stands (C) during 2003-2008.

During the first three years after the tornado (2003-2005), a relatively small decrease of about 20.2 mg in MIB was observed in the disturbed stands (from 306.7 mg to 286.5 mg) and about 55.1 mg in the control stands (from 388.0 mg to 332.9 mg; Fig. 3). In 2006, MIB was 134.0 mg lower in disturbed than in control stands (149.4 mg *vs.* 283.4 mg; LSD test, p < 0.001). The largest difference in MIB between disturbed and control stands was in 2008 (121.1 mg *vs.* 260.8 mg; LSD test, p < 0.001; Fig. 3).

Considering the entire study period, the SPC values were on average 200.0 in the disturbed and 280.0 in the control stands (LSD test, p < 0.001; Table 2). In 2006, the difference in SPC between disturbed and control stands was 111.1 units (180.5 *vs.* 291.5; LSD test, p < 0.001). The highest difference was 128.8 units in 2008 (268.8 *vs.* 144.0; LSD test, p < 0.001). Both ANOVA and LSD test confirmed the reduction of MIB and SPC in the disturbed stands, compared with the control stands (Table 2).

**Figure 4.**
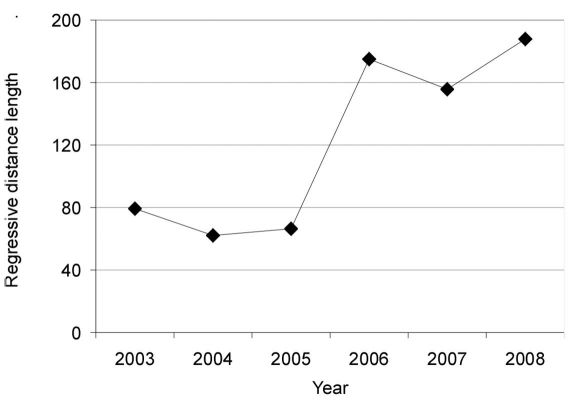
Regression distances between carabid assemblages inhabiting post-tornado (disturbed) and control stands during 2003-2008.

In the first year after the tornado (2003), the mean regression distance of carabid assemblages between disturbed and control stands was 79.0 ± 47.9 (Fig. 4). In 2004 and in 2005, the regression distances were 61.8 ± 44.0 and 66.2 ± 35.4, respectively. The regression distance peaked in 2006, being 174.8 ± 28.1 (LSD test, p < 0.001), and remained high until 2008 (188.1 ± 29.4).

## Discussion

The environment under study had been subject to a major disturbance that severely affected carabid assemblages in the impacted stands: a tornado. During the first few years after the disturbance, soil weight humidity was 1.6% lower in the disturbed than in the control stands; moreover, soil nitrogen concentration was higher, and the lack of tree crowns that usually acidify the rain water may have led to an increase in the soil pH (Skłodowski 2007a). The soil of the disturbed stands, compared to the control stands, was characterized by a lower rate of soil CO2 diffusion and lower decomposition rate of organic matter (Skłodowski 2007a). In the disturbed stands, mosses and dwarf shrubs may consequently have suffered: *Pleurozium schreberi*, *Dicranum* spp. and *Vaccinium myrtillus*. However, the percent covers of *Vaccinium vitis-idaea* and *Deschampsia flexuosa* – species favored by an increased amount of light and soil nitrogen supply (Skłodowski and Buszyniewicz 2007; Sławski 2007) – had increased. The above characteristics suggest drastic changes in environmental conditions of the disturbed stands. These changes were in turn accompanied by alterations in the carabid assemblage structure.

During the entire six-years period following the tornado impact, carabid richness was 33% higher in disturbed than in control stands (Table 1). This finding is in agreement with several studies done in tornado-impacted stands (Otte 1989a, 1989b; Bouget and Duelli 2004; Bouget 2005c; Gandhi et al. 2008). Despite this higher carabid species richness, carabids were generally less abundant in the disturbed stands for the second and third post-tornado summers. Similarly, Bouget (2005c) and Gandhi et al. (2008) observed up to two times lower catch rates in tornado-impacted than in adjacent intact forests 2-4 years after the tornado. As late as post-tornado years 5 and 6, species richness became higher in the disturbed than in the control stands (Table 1), which suggests a delayed response for a tree-fall caused increase in openness and micro-climatic changes. During the first three years after the tornado (2003-2005), some forest species declined (e.g., *Carabus violaceus*) while non-forest species had not yet started to colonize the tornado-impacted forests. Many non-forest species appeared in the fourth post-tornado year, such as *Amara aenea*, *Amara communis*, *Amara equestris*, *Amara lunicollis*, *Harpalus flavescens*, *Harpalus solitaris* and *Microlestes minutulus*.

Generally, carabid species richness is positively influenced by disturbances in forest ecosystems, such as wildfire (e.g., Szyszko 1990; Skłodowski 1995; Fernández Fernández and Saldago Costas 2004; Buddle et al. 2006; Koivula and Spence 2006; Paquin 2008) and clear-cutting (e.g., Szujecki et al. 1983; Szyszko 1983, 1990; Niemelä et al. 1993; Haila et al. 1994; Skłodowski 1995, 1997, 2006a, 2006b, 2008, 2009; Koivula 2002a, 2002b; Koivula et al. 2002; du Bus de Warnaffe and Lebrun 2004; Huber and Baumgarten 2005; Martikainen et al. 2006). An increase in carabid species richness in disturbed forest ecosystems is one consequence of the transformation of an earlier structurally homogenous forest stand (only one or a few tree species with even age structure of dominant trees) into a mosaic of smaller sub-patches that have been impacted by the disturbance to varying degrees (Otte 1989a, 1989b; Niemelä et al. 1996, 2007; Koivula et al. 2002; Bouget and Duelli 2004; Bouget 2005c; Koivula and Spence 2006; Pearce and Venier 2006; Ulyshen et al. 2006; Gandhi et al. 2008; Paquin 2008). Open gaps become rapidly colonized by open-area and eurytopic species (Koivula 2002a, 2002b); besides, also most forest species may survive (Buddle et al. 2006; Koivula and Spence 2006). Because the rate of immigration by species not associated with closed canopy is higher than the rate of decline of canopy-closure specialists, the total number of species increases, at least in the short term (Niemelä et al. 1988, 1993, 2007; Koivula 2002a, 2002b; Bouget 2005c; Huber and Baumgarten 2005).

The decrease in epigeic carabid catch rates can be related with the development of forest floor vegetation (Skłodowski 2002, Poole et al. 2003, Sroka and Finch 2006, Taboada et al. 2006).

Another explanation could be the fact that the decline of forest species in the disturbed stands was not accompanied by the appearance of non-forest species during the first three years after the tornado. The disturbed stands started to be colonized by eurytopic and open-area species (particularly by *Amara lunicollis*) as late as in 2006, which resulted in an increase in the total catch rate. Yet another explanation might be obtained from weather conditions. Many indices peaked in 2006. Over the entire period of study, that year was characterized by cold winter air temperature (-32,2ºC near the ground) and high summer temperature (maximum air temperature +32,6ºC) accompanied by the lack of precipitation (data from the Hydrologic-Meteorological Station of Olsztyn; Biuletyn Państwowej Służby Hydrologiczno-Meteorologicznej IMiGW).

During 2007-2008, carabids with an European range, including western Siberia, decreased (Fig. 1). Similar changes have previously been associated with other disturbances, such as clear-cutting and wildfire (Szyszko 1983, 1990; Skłodowski 1995, 1997). In the disturbed stands, a decrease of forest species (including specialists and succession generalists) was observed. In the last study year these species contributed to about 46% of all carabids in disturbed stands in comparision to 96% in the control (Table 1), which is in line with (Otte (1989a, 1989b), Bouget and Duelli (2004), Bouget (2005c) and Gandhi et al. (2008). Eurytopic species, characterized by high tolerance against changing environmental conditions, slowly appeared in the post-tornado stands. Niemelä et al. (1993) and Koivula et al. (2002) found that clear-cutting may not significantly influence the frequency of eurytopic species during 2-3 years after the disturbance. However, in the tornado-disturbed stands, studied here, the proportion of euryptopic species increased significantly. This increase was associated with a comparable decline of forest carabids and contributed to the elevated catch rate of eurytopic carabids and consequently total species richness in 2006-2008 (see also Niemelä et al. 1993, 1996, 2007; Skłodowski 1995, 1997, 2002; Bouget 2005c; for clear-cut areas, see Szujecki et al. 1983; Szyszko 1983, 1990; Koivula et al. 2002; Elek et al. 2005; Huber and Baumgarten 2005; for post-fire forests, see Fernández Fernández and Saldago Costas 2004; Pearce and Venier 2006; Ulyshen et al. 2006).

The delayed tornado response of three years by carabids was most clearly seen in the decreasing catches of both large and small zoophages and an accompanying, up to 45.5%, increase in the share of hemizoophages (Table 1). At the beginning of the present study, large zoophages dominated the carabid assemblages in both the tornado-impacted and the control stands , but since 2006 the impacted stands became dominated by hemizoophages, particularly *Amara lunicollis*. Otte (1989a) and Gandhi et al. (2008) recorded an appearance of hemizoophagous *Amara* and *Harpalus* species two years after the tornado. Similarly, Bouget (2005c) found that carabid species characteristic for post-tornado stands are hemizoophages, such as *Amara plebeja* and *Amara similata*. The increase of hemizoophages and the decrease of large zoophages are general patterns following a disturbance, such as clear-cutting and wildfire (Szujecki et al. 1983; Szyszko 1983, 1990; Skłodowski 1995, 1997, 2006a, 2006b). The colonization of *Deschampsia flexuosa* in the tornado-impacted stands (Skłodowski and Buszyniewicz 2007; Sławski 2007) may favor seed-eating hemizoophages, such as *Amara lunicollis*, *Harpalus rufipalpis* and *Harpalus rufipes*.

In the tornado-impacted stands, brachypterous species declined while macropterous species associated with arid, sunny, grassy habitats increased (Burakowski et al. 1973, 1974; Niemelä et al. 1993; Koivula 2002a; Koivula et al. 2002; Koivula and Niemelä 2003; Huber and Baumgarten 2005). This tendency became particularly clear in 2006 (Table 1) and is in line with Otte (1989a), Du Bus, de Warnaffe and Lebrun (2004), Bouget (2005c) and Gandhi et al. (2008). Similar observations have been made in post-fire areas (Fernández Fernández and Saldago Costas 2004; Skłodowski 1995), in clear-cuts (Szujecki et al. 1983; Szyszko 1983, 1990; Skłodowski 1995, 1997, 2006a, 2006b; Butterfield 1997; du Bus de Warnaffe and Lebrun 2004; Elek et al. 2005), and in clear-cuts with prescribed fire (Martikainen et al. 2006).

The species composition of tornado-impacted stands was different in impacted and in control stands. Similar decreases in species similarity in carabid assemblages between post-tornado and control stands have been reported by Otte (1989a) and Gandhi et al. (2008). The similarity of carabid assemblages between disturbed and control stands decreased with time: the lowest similarity was recorded in 2007. In the subsequent years the faunistic differences became more pronounced due to the species-exchange process and to the changing dominance structure of the assemblages inhabiting the disturbed ecosystem. On the other hand, the observed increase in the compositional similarity along with an increase in the structural dominance in 2008 suggests a slowing-down of the tornado-caused changes in the carabid assemblages.

The effect of the tornado on forest carabids was best illustrated by the MIB and SPC indices (Fig. 3). The decrease in MIB in the control stands throughout the study may have resulted from an increasing proportion of the small zoophage *Calathus micropterus* and proportional decreases of the larger *Carabus arvensis* and *Carabus violaceus*. The pattern was different in the disturbed stands where both indices used for the construction of the SPC/MIB model had been continuously decreasing, visualized by their gradual movement towards the bottom-and-left-hand corner in Fig. 3. Such a pattern suggests an enhanced regression (changes in structure and functioning of assemblages from higher to lower levels of succession development) of carabid assemblages and their habitat after disturbance (Skłodowski 1995, 1997, 2009). The unequal rate of change of indices between disturbed and control stands is worth noting. During 2003-2005, MIB and SPC marginally decreased in the tornado-impacted stands, and the regression changes, as expressed in the length of the regression distance, seemed not to grow (Fig. 4). A shorter regression distance means smaller differences in the structure and functioning of carabid assemblages between disturbed and control stands. Based on the SPC index alone, one may come to the conclusion that the carabid assemblages inhabiting post-tornado stands in 2005 may be considered typical for 30-70-years old stands (as predicted in a calibrated SPC/MIB model; see Skłodowski 1995, 1997, 2009). In 2006 the loss of carabids associated with mature, undisturbed stands is also worth noting: the decline of species with an autumn development, European, and big forest zoophages species also influenced the decrease of SPC and the regression distance more than doubled, suggesting increasingly pronounced changes in the structure and functioning of carabid assemblages (Fig. 3). Until 2008, no significant changes in the direction of succession in the tornado-damaged stands were observed; as a matter of fact, during 2006-2008 the rate of faunal change slowed down, indicated by only slight changes in MIB, SPC and the regression distances. A decrease in SPC, observed especially in the sixth year after the tornado, was equivalent with a change of carabid fauna to a phase typical for 3-10-years old Scots pine stands (the calibrated SPC/MIB model; Skłodowski 1995, 1997).

The most important change in the carabid assemblages of tornado-disturbed stands was the change toward a species composition typical for early-successional, regenerating forests: carabids associated with late-successional forests were partly replaced by eurytopic and pioneer species characteristic of early successional phases. The chronology of the observed successional changes is particularly interesting. The most drastic change in the carabid fauna takes place during the first three years following a disturbance (Szyszko 1990, Koivula et al. 2002). In the present study, however, changes in the post-tornado stands really accelerated 4-6 years after the tornado impact, which suggests that the most drastic alterations in carabid assemblage, caused by the tornado, are delayed by 4-6 years. Besides, based on the present results it is reasonable to assume that faunal changes will continue and possibly become more pronounced more than six years after the disturbance event, even though the rate of change may be slower. It has to be emphasized that no signs of recovery of the impacted carabid assemblages were observed during this study.

This six-year study demonstrated a long-lasting down-turn of the forest carabid assemblage, suggesting a substantial change in this environment. Field data collected in the seventh year after the tornado impact, that is 2009, are subject to analyses. Even though these data have been only preliminarily elaborated so far, it seems that the carabid communities have eventually started to recover. Among other signals of recovery, forest species seem to increase in abundance, and the values of MIB and SPC are also increasing, and the regression distance between disturbed and control stands has decreased. Following the emergence of new, naturally regenerated seedling trees (apart from 2-3 years old pine and birch) one can expect that the subsequent years will be characteristic of both the recovery of forest-carabid fauna, and the recovery of the entire ecosystem. It is noteworthy that in the Pisz Forest the trees that survived are not threatened by under-the-bark pests, due to the fact that the hurricane disturbance of the forest had taken place in the month of July, thus disrupting the main season of these pests� flight and the period of egg lying, a yearly spring event. Moreover, in the subsequent years, the injured trees became dry and did not attract dead-wood dependent insects any more. Therefore, a reasonable suggestion is to retain some fallen trees in the tornado-impacted stands and wait for the spontaneous natural regeneration of the forest ecosystem.

## Appendix A

The most abundant carabid species and their participation in communities inhabiting disturbed stands and controls stands during observations performed in years: 2003-2008.

**Table:**

Control		Disturbed	
2003			
*Carabus arvensis*	24.4 ± 15.1%	*Pterostichus niger*	24.7 ± 12.4%
*Carabus violaceus*	25.4 ± 15.2%	*Carabus arvensis*	20.0 ± 11.6%
*Pterostichus oblongopunctatus*	12.4 ± 9.5%	*Pterostichus oblongopunctatus*	23.3 ± 8.8%
*Calathus micropterus*	11.9 ± 8.8 %	*Carabus violaceus*	16.8 ± 10.1%
*Pterostichus niger*	7.7 ± 5.5%	*Carabus coriaceus*	7.1 ± 5.2%
2004			
*Carabus arvensis*	31.7 ± 15.8%	*Pterostichus oblongopunctatus*	29.6 ± 8.7%
*Pterostichus oblongopunctatus*	19.6 ± 12.3%	*Carabus arvensis*	24.9 ± 12.8%
*Carabus violaceus*	19.5 ± 12.4%	*Pterostichus niger*	20.3 ± 8.5%
*Pterostichus niger*	6.8 ± 5.1%	*Carabus violaceus*	12.2 ± 6.5%
		*Carabus coriaceus*	5.4 ± 3.3%
2005			
*Carabus arvensis*	43.9 ± 15.1%	*Carabus arvensis*	22.3 ± 9.0%
*Carabus violaceus*	20.5 ± 11.1%	*Pterostichus niger*	21.8 ± 7.9%
*Pterostichus niger*	6.4 ± 4.6%	*Calathus micropterus*	14.5 ± 13.0%
*Pterostichus oblongopunctatus*	7.6 ± 5.4%	*Pterostichus oblongopunctatus*	16.8 ± 7.0%
*Amara lunicollis*	6.6 ± 5.8%	*Carabus violaceus*	10.6 ± 5.4%
*Calathus micropterus*	5.7 ± 3.9%	*Carabus coriaceus*	8.3 ± 4.2%
			
2006			
*Carabus arvensis*	31.7 ± 23.9%	*Pterostichus niger*	27.3 ± 9.4%
*Calathus micropterus*	15.2 ± 11.6%	*Calathus micropterus*	22.2 ± 10.1%
*Amara lunicollis*	12.7 ± 12.2%	*Carabus arvensis*	17.4 ± 6.5 %
*Carabus violaceus*	7.1 ± 5.0%	*Pterostichus oblongopunctatus*	14.4 ± 4.3%
		*Carabus violaceus*	8.6 ± 3.0%
2007			
*Carabus arvensis*	31.4 ± 22.2%	*Pterostichus niger*	30.5 ± 12.4%
*Amara lunicollis*	22.9 ± 14.3%	*Calathus micropterus*	25.5 ± 12.1%
*Calathus micropterus*	8.4 ± 3.7%	*Carabus arvensis*	15.1 ± 6.7%
*Pterostichus niger*	9.1 ± 7.4%	*Pterostichus oblongopunctatus*	13.2 ± 4.8%
*Harpalus rufipalpis*	5.0 ± 3.3%	*Carabus violaceus*	7.8 ± 4.1%
2008			
*Amara lunicollis*	36.7 ± 18.7%	*Carabus arvensis*	26.1 ± 11.5%
*Carabus arvensis*	17.2 ± 11.0%	*Pterostichus niger*	20.2 ± 10.0%
*Calathus micropterus*	8.7 ± 5.7%	*Pterostichus oblongopunctatus*	16.4 ± 6.4%
*Pterostichus niger*	10.4 ± 8.1%	*Calathus micropterus*	15.0 ± 9.3%
*Carabus violaceus*	5.1 ± 3.8%	*Carabus violaceus*	11.8 ± 3.4%
			

## Appendix B

The changes of dominance indices of some of the most abundant carabid species inhabiting disturbed stands D and controls stands C.

**Table:**

*Species*	*Stand type*	*2003*	*2004*	*2005*	*2006*	*2007*	*2008*
*Amara lunicollis* (Schiodte 1837)	D	1.15	2.57	6.63	12.66	22.92	36.69
	C	0	0.04	0.06	0.19	0.28	1.2
*Calathus erratus* (C.R. Sahlberg 1827)	D	3.07	1.09	0.31	3.29	3.79	5.1
	C	0.29	0.2	0.18	0.09	0.06	0.52
*Calathus micropterus* (Duftschmid 1812)	D	11.95	2.96	5.8	15.21	8.38	8.79
	C	2.89	2.28	14.52	22.28	25.49	15.01
*Carabus arvensis* Herbst 1784	D	24.36	31.68	43.89	31.68	31.44	17.15
	C	19.99	24.94	22.32	17.39	15.11	26.14
*Carabus coriaceus* (Linnaeus 1758)	D	0	0	0	0	0	0.01
	C	7.13	5.39	8.38	4.93	2.88	2.74
*Carabus hortensis* Linnaeus 1758	D	1.09	1.54	0.81	0.17	0.26	0.14
	C	0.73	1.12	0.86	0.85	0.81	0.51
*Carabus violaceus* Linnaeus 1758	D	25.37	19.56	20.48	7.06	4.45	5.14
	C	16.79	12.2	10.66	8.6	7.89	11.75
*Harpalus rufipalpis* Sturm 1818	D	0.31	0.33	0.41	3.44	4.96	3.22
	C	0	0	0	0.02	0.02	0
*Pseudoophonus rufipes* (Degeer 1774)	D	0.85	0.6	1.34	3.81	3.92	1.74
	C	0	0	0	0.01	0.02	0.02
*Pterostichus niger* (Schaller 1783)	D	7.71	6.84	6.41	3.64	9.11	10.38
	C	24.67	20.32	21.8	27.31	30.53	20.2
*Pterostichus oblongopunctatus* (Fabricius 1787)	D	12.41	19.61	7.63	4.28	1.69	1.89
	C	23.34	29.65	16.84	14.38	13.25	16.39
